# Non-Targeted Metabolomics Reveals the Effects of Different Light Cycles on *Samsoniella hepiali*

**DOI:** 10.3390/biology14121745

**Published:** 2025-12-05

**Authors:** Chao Feng, Hao-Xu Tang, Bing Jia, Jin-Xuan Yan, Xiu-Zhang Li, Yu-Ling Li

**Affiliations:** State Key Laboratory of Plateau Ecology and Agriculture, Qinghai Academy of Animal and Veterinary Science, Qinghai University, Xining 810016, China; 18667661285@163.com (C.F.); haoxutang0717@163.com (H.-X.T.); bingjia0415@163.com (B.J.); yanjinxuan2666@163.com (J.-X.Y.)

**Keywords:** *Samsoniella hepiali*, antioxidant activity, non-targeted metabolomics, KEGG enrichment

## Abstract

This study aimed to investigate the effects of different light treatments on the growth, antioxidant activity, and metabolite profiles of *Samsoniella hepiali*. Mycelial biomass, bioactive components, antioxidant capacity, and metabolomic profiles were analyzed under dark, continuous light, and 12 h light/12 h dark cycle conditions. The results showed that the 12 h light/dark cycle significantly reduced dry mycelial weight compared to the dark and continuous light groups (*p* < 0.001), while no significant difference in biomass was observed between the latter two. Polysaccharide (Pol) content did not differ significantly among the three groups. In terms of antioxidant activity, the continuous light group exhibited the highest protein (Prot) content, total phenolic (TP) content, DPPH radical scavenging activity, and ferric reducing antioxidant power (FRAP) as well as the lowest superoxide anion (·O_2_^−^) content. The dark group showed the highest activities of superoxide dismutase (SOD) and catalase (CAT). Correlation analysis revealed that total phenolic (TP) content was significantly positively correlated with 2,2-Diphenyl-1-picrylhydrazy (DPPH) and FRAP (*p* < 0.01), and significantly negatively correlated with superoxide anion content (*p* < 0.05). Non-targeted metabolomics identified 3643 metabolites, primarily amino acids and derivatives, organic acids, and glycerophospholipids. KEGG enrichment analysis indicated significant accumulation of differential metabolites such as linoleic acid, tyrosine, and phosphatidylcholine across comparison groups. These findings provide insights into the regulatory role of light exposure on the antioxidant capacity of *S. hepiali* and support its further development in fermented mycelial products.

## Simple Summary

Light is a powerful environmental signal that can influence the growth and metabolism of fungi. In this study, we investigated how different light exposures—constant darkness, a 12 h daily light cycle, and constant light—affect the medicinal fungus *Samsoniella hepiali*. We found that constant light resulted in the strongest antioxidant profile, showing the highest peroxidase (POD) activity, 2,2-Diphenyl-1-picrylhydrazy(DPPH) radical scavenging rate, ferric reducing antioxidant power (FRAP) value, and the lowest superoxide anion (·O_2_^−^) content, along with the highest levels of proteins (Prot) and total phenolics (TP). In contrast, the 12 h light cycle reduced fungal growth but triggered the highest hydroxyl radical (·OH) scavenging rate. Our analysis of over 3000 metabolites revealed that light reshapes the fungus’ metabolism, enriching key pathways linked to these antioxidant traits. These results demonstrate that manipulating light conditions represents a promising approach to simultaneously enhance the medicinal properties and market potential of *Samsoniella hepiali*.

## 1. Introduction

*Ophiocordyceps sinensis* (Berk.) G.H. Sung, J.M. Sung, Hywel-Jones and Spatafora, a prized traditional Chinese medicine (TCM), has been documented for over one millennium. It is an entomopathogenic complex consisting of the stroma of the ascomycetous fungus and the cadaver of a bat-moth larva that the fungus parasitizes [1,2]. Colloquially, the “winter worm” denotes the fungus-killed larva, whereas the “summer grass” refers to the fungal stroma emerging from the insect’s head. The species is endemic to the Qinghai–Tibet Plateau and the adjoining Hengduan Mountains, and TCM attributes to it the efficacy of replenishing lung and kidney, arresting hemorrhage, resolving phlegm, and relieving asthma. Together with ginseng and deer antler it is venerated as one of the “Three Treasures of China [3,4,5]”.

The sustainable utilization of *O. sinensis* faces significant challenges due to resource depletion caused by overharvesting and the technical-economic constraints of artificial cultivation. In this context, biotechnological utilization of its anamorphic stage, *S. hepiali*, through fermentation processes has emerged as a viable strategy to address resource scarcity. *S. hepiali* demonstrates substantial phylogenetic and metabolic relationships with its teleomorph, *O. sinensis*. This fungus was initially isolated from *O. sinensis* specimens collected in the Baima Snow Mountain region of Yunnan Province by Professor Dai Ruqin’s research team, with patent protection granted in 1993. As the principal anamorph of *O. sinensis*, *S. hepiali* produces comparable bioactive compounds during fermentation, including adenosine, polysaccharides, and mannitol—key pharmacological components characteristic of the natural fungus. Scientific evidence confirms that fermented products of *S. hepiali* exhibit pharmacological activities parallel to wild *O. sinensis*, particularly in antioxidant capacity and immunomodulatory effects, providing scientific rationale for its substitutive applications [6,7]. Fermentation technology represents a paradigm shift from reliance on scarce wild resources to sustainable production, not only alleviating pressure on natural populations but also enabling standardized preparation of *O. sinensis*-associated bioactive compounds. Commercial products developed from *S. hepiali* strains have been established as substantiated alternatives to wild-collected *O. sinensis* and are widely employed in clinical practice [6,7,8,9,10,11].

*S. hepiali* exhibits a chemical and pharmacological profile comparable to that of *O. sinensis* [8]. Its major bioactive constituents include Cordyceps polysaccharides composed predominantly of galactose, glucose, and arabinose, which exert antitumor and antiviral activities [9,10]. Nucleosides (adenosine, cordycepin, cytidine, guanosine, and uridine) that display antithrombotic, neuroprotective, immunomodulatory, and antineoplastic properties, involving Succinate metabolism and its regulation of host–microbe interactions [11]. D-mannitol (cordycepic acid) elicits diuretic, intracranial-pressure-lowering, and nephroprotective effects. Sterols, a family encompassing 141 compounds including ergosterol, β-sitosterol, and cholesterol [11], which contribute anti-inflammatory, antimicrobial, and anticancer actions [12]. Additionally, there are proteins and amino acids associated with anti-fatigue activity [13].

Unlike plants, fungi are generally considered incapable of photosynthesis and must rely on extracellular enzymes to break down cultivation substrates to obtain nutrients for mycelial growth and fruiting body formation. In other words, fungi utilize light primarily as an informational signal rather than an energy source. Light exposure plays a crucial role in promoting primordium differentiation in higher fungi, and significantly influences the formation, coloration, morphology, yield, and metabolic production of fruiting bodies. Light functions as a crucial environmental cue that orchestrates fungal development, secondary metabolism, and morphogenesis. Blue light, for example, is perceived by photoreceptors such as the White collar complex, which subsequently activates the cAMP/PKA cascade to modulate hyphal polar growth and conidiation [14]. Conversely, UV or high-intensity light can impose oxidative stress, thereby redirecting metabolic fluxes. Previous studies on *Ophiocordyceps* and related taxa indicate that light quality, intensity, and photoperiod markedly affect colony morphology, biomass, and metabolite accumulation [15]. However, the reported effects are inconsistent. While some data indicate that blue light stimulates cordycepin biosynthesis, others demonstrate that continuous darkness favors polysaccharide production, underscoring species- and even strain-specific responses [16,17,18,19,20,21]. Numerous studies have revealed the species-specific regulatory effects of light quality. For instance, red light is generally considered beneficial for mycelial growth and fruiting body morphogenesis. Research indicates that red light is most conducive to the mycelial growth of *Panus similis.* for *Pleurotus eryngii*, red light (628 nm) has been identified as the optimal light quality for primordium formation and development. In contrast, the effects of blue light are more complex. In the cultivation of *Auricularia heimuer* and *Ganoderma lucidum*, blue light or dark conditions favor rapid mycelial growth, and the mycelial morphology under blue light is more robust. However, under liquid fermentation conditions, blue light significantly inhibits the mycelial growth of *Cordyceps militaris*. Beyond red and blue light, yellow light has been demonstrated to strongly induce primordium formation in *Panus similis*, while white/natural light has been shown to promote mycelial growth and improve the commercial quality of fruiting bodies in *Pleurotus ostreatus* and *Pleurotus djamor* [22,23,24,25,26,27,28]. In terms of metabolic regulation, the role of blue light is particularly prominent. Studies have confirmed that blue light treatment effectively increases the diversity and content of triterpenic acids in *Ganoderma lucidum* mycelia and significantly enhances the accumulation of polysaccharides at various developmental stages. In *Cordyceps militaris*, blue light favors the accumulation of cordycepin in fruiting bodies and markedly elevates the carotenoid content in mycelia. Furthermore, exposure of *Hypsizygus marmoreus* fruiting bodies to LED blue light increases their ergosterol and total polyphenol contents, consequently enhancing their antioxidant capacity [23]. In addition to light quality, precise regulation of photoperiod and light intensity is equally indispensable for achieving high-efficiency production in edible fungi. Optimized photoperiod strategies can significantly enhance biomass and resource utilization efficiency. Research has found that intermittent exposure of *Pleurotus geesteranus* to LED light on a 12 h light/12 h dark cycle not only promotes mycelial growth but also improves its degradation of selenium-enriched wheat bran and the conversion of selenium nutrients [29]. For *Lentinula edodes*, an efficient miniaturized lighting protocol demonstrates that daily exposure to just 1 min of LED green light at 0.4 W/m^2^ can increase mycelial biomass by 50–100%, offering a new approach for cost reduction and efficiency improvement in industrial production [30]. In the cultivation of *Pleurotus ostreatus*, studies have determined that a light intensity of 500–700 lux with a daily photoperiod of 14 h yields maximum production [31]. Moreover, tailored light supplementation strategies based on specific light qualities and photoperiods have been developed for different varieties of *Flammulina velutipes*, effectively avoiding resource waste and adverse effects associated with non-selective light supplementation [32,33].

Although the genetic background and fermentation processes of *S. hepiali* have been extensively documented, the molecular mechanisms by which light regulates its growth-related gene expression remain largely unexplored. Industrial fermentation protocols currently rely on darkness, and systematic investigations of photic environments are lacking. Therefore, the present study aims to elucidate the impact of photoperiod on the growth and metabolism of *S. hepiali* by integrating physiological analyses with untargeted metabolomics, thereby providing mechanistic insights for developing efficient, stable, and controllable light-regulated fermentation strategies.

## 2. Materials and Methods

### 2.1. Reagents

HPLC-grade acetonitrile and methanol were purchased from Merck (Darmstadt, Germany). Milli-Q water was supplied by Millipore (Bradford, Burlington, MA, USA). Ammonium acetate and formic acid were obtained from Sigma-Aldrich (St. Louis, MO, USA); the standards were stored at −20 °C in the dark.

### 2.2. Sample Origin

The *Samsoniella hepiali* strain was isolated from wild *Ophiocordyceps sinensis* collected in Henan County, Qinghai Province, China (101°36′19.0″ E, 34°44′8.3″ N; 3600 m).

### 2.3. Medium Preparation

Seed medium: 15% potato extract, 0.2%peptone, 3%glucose, 0.2%KH_2_PO_4_, 0.02%MgSO_4_. PDA medium: 20% potato extract, 0.3% peptone, 2% glucose, 0.2% KH_2_PO_4_, 0.02% MgSO_4_. Preparation of potato culture medium: First, the peeled and weighed potato tubers were diced and boiled in distilled water. The mixture was brought to a boil and then simmered for 30 min. Finally, the solution was filtered through four layers of sterile gauze, and the filtrate was collected for subsequent use. The culture medium was modified [34].

### 2.4. Mycelium Harvest

*Samsoniella hepiali* was inoculated into seed medium and activated at 23 °C for 4 d on a rotary shaker (ZQZY-AGS8, Zhichu, Shanghai, China). The seed culture was then transferred to PDA medium and incubated at 23 °C under 0 h (CK), 12 h or 24 h illumination on a rotary shaker (full-spectrum plant growth LEDs, 160 lx) for 7d (ZQZY-BGS8, Zhichu, Shanghai, China). The lighting was provided from the top using energy-saving LED panels, ensuring uniform illumination at all points within the chamber. The distance between the light source and the samples was consistently maintained at 15 cm to guarantee identical light exposure for all samples. The heating interference caused by the light intensity in the incubator was minimal, with a temperature increase in less than 0.5 °C, which was negligible and considered unlikely to introduce significant experimental error.

Other conditions included the use of 250 mL Erlenmeyer flasks (Shuniu, Chongzhou China), each containing 150 mL of liquid medium. The shaker in the incubator was set to a rotational speed of 135 RPM; The sample was centrifuged in a bench-top low-temperature centrifuge (Hesion, Changsha, China), filtered to collect the mycelia, and stored at −80 °C (Haier, Qingdao, China) until analysis. Each light treatment had three biological replicates.

### 2.5. Determination of Bioactive Compounds and In Vitro Antioxidant Activity

Antioxidant activity over time is typically monitored by measuring the levels of DPPH, hydroxyl radical (·OH), FRAP, CAT, POD, SOD, and superoxide anion (·O_2_^−^). When antioxidant molecules react with free radicals, the absorbance of the system decreases, and the rate of change in absorbance serves as an indicator of antioxidant capacity. The kits used in this study were purchased from Suzhou Keming Biotechnology Co., Ltd. (Suzhou, China), and the detection methods followed the instructions provided with the kits and relevant references [30,31].

The absorbance for DPPH radical scavenging capacity was measured at 515 nm.

The absorbance for ·OH radical scavenging activity was measured at 550 nm.

The FRAP assay is a new method for evaluating “antioxidant capacity”. It is low-cost, simple in reagent preparation, and has high result reproducibility. The absorbance for FRAP radical scavenging capacity was measured at 593 nm.

The catalytic activity of CAT can be reflected by measuring the absorbance of the remaining hydrogen peroxide in the system at 405 nm.

Superoxide anions (·O_2_^−^) are generated through the xanthine-xanthine oxidase reaction system. ·O_2_^−^ can reduce nitroblue tetrazolium to form blue formazan, which has an absorption at 560 nm; SOD can scavenge ·O_2_^−^, thereby inhibiting the formation of formazan. The darker the blue color of the reaction solution, the lower the SOD activity, and vice versa. SOD activity was determined by measuring the absorbance at 560 nm.

POD catalyzes the oxidation of specific substrates by H_2_O_2_, which has a characteristic light absorption at 470 nm.

Superoxide anions react with hydroxylamine hydrochloride to form NO_2_^−^, which then reacts with sulfanilic acid and α-naphthylamine to generate a red azo compound with a characteristic absorption peak at 530 nm.

Total polysaccharides (Pol) were extracted using the water extraction and alcohol precipitation method, and their content was determined by the phenol-sulfuric acid method with absorbance measured at 490 nm.

Under alkaline conditions, cysteine, cystine, tryptophan, tyrosine, and peptide bonds in protein can reduce Cu^2+^ to Cu^+^; 2 molecules of BCA combine with Cu^+^ to form a purple complex, which has an absorption peak in the range of 540–595 nm, with the strongest absorption at 562 nm.

Under alkaline conditions, phenolic substances (TP) reduce tungstomolybdic acid to produce a blue compound with a characteristic absorption peak at 760 nm. The total phenol content of the sample can be obtained by measuring the absorbance at 760 nm.

### 2.6. Fresh Sample Extraction

The fresh sample and steel beads were placed in the grinding jar and capped. Pre-cool the spoon and grinding jar in liquid nitrogen until the liquid nitrogen no longer boils. Grind (30 Hz, 30 S) to powder form using a grinder (MM400, Retsch, Leipzig, Germany). Weigh 50 mg of the sample using an electronic balance (MS105DΜ, Mettler-Toledo GmbH, Zurich, Switzerland), press the confirmation button to save the weighing result, and weigh all samples one by one until the project is completed. Add 600 μL of 70% methanol with internal standard extract to the sample (add 600 μL of 70% methanol with internal standard extract per 50 mg for samples less than 50 mg). Vortex once for 30 s each time for a total of 6 times. Allow to stand for 30 min at −20 °C in the refrigerator. After centrifugation (12,000 rpm, 3 min), the supernatant was aspirated, and the sample was filtered through a microporous membrane (0.22 μm pore size) and stored in the injection vial for UPLC-MS/MS analysis.

#### 2.6.1. HPLC Conditions

All samples were acquired by the LC-MS system following machine orders. The analytical conditions were as follows: UPLC: column, Waters ACQUITY UPLC HSS (Waters, Shanghai, China) T3 1.8 µm, 2.1 mm ×100 mm; column temperature, 40 °C; flow rate, 0.40 mL/min; injection volume, 4 μL; solvent system, water (0.1% formic acid): acetonitrile (0.1% formic acid); Sample measurements were performed with a gradient program that employed the starting conditions of 95% A, 5% B. Within 5 min, a linear gradient to 35% A, 65% B was programmed. Within 1 min, a linear gradient to 1% A, 99% B was programmed, kept for 1.5 min. Subsequently, a composition of 95% A, 5.0% B was adjusted within 0.1 min and kept for 2.4 min.

#### 2.6.2. MS Conditions (QE)

All the methods alternated between full scan MS and data dependent MSn scans using dynamic exclusion. MS analyses were carried out using electrospray ionization in the positive ion mode and negative ion mode using full scan analysis over *m*/*z* 84–1250 at 35,000 resolution. Additional MS settings are ion spray voltage, 3.5 KV or 3.2 KV in positive or negative modes, respectively; Sheath gas (Arb), 30; Aux gas, 5; Ion transfer tube temperature, 320 °C; Vaporizer temperature, 300 °C; Collision energy, 30, 40, 50 V; Signal Intensity Threshold, 10 × 10^−5^ cps; Top N vs. Top speed, 10; Exclusion duration, 3 s.

### 2.7. Data Analysis

#### 2.7.1. PCA

Unsupervised PCA (principal component analysis) was performed by statistics function prcomp within R (www.r-project.org, accessed on 15 March 2025). Before unsupervised PCA, the preprocessing methods are log^2^ transform and mean centering.

#### 2.7.2. Hierarchical Cluster Analysis and Pearson Correlation Coefficients

The HCA (hierarchical cluster analysis) results of samples and metabolites were presented as heatmaps with dendrograms, while Pearson correlation coefficients (PCC) between samples were calculated by the core function in R and presented as only heatmaps. Both HCA and PCC were carried out by R.4.5.2. For HCA, normalized signal intensities of metabolites (unit variance scaling) are visualized as a color spectrum.

#### 2.7.3. Differential Metabolites Selected

For two-group analysis, differential metabolites were determined by VIP (VIP > 1) and absolute Log2FC (|Log2FC| ≥ 1.0). VIPs were extracted from OPLS-DA result, which also contain score plots and permutation plots; the result was generated using R.4.4.2. The data were log transform (log2) and mean centering before OPLS-DA. In order to avoid overfitting, a permutation test (200 permutations) was performed.

#### 2.7.4. KEGG Annotation and Enrichment Analysis

Identified metabolites were annotated using KEGG Compound database (http://www.kegg.jp/kegg/compound/, accessed on 15 March 2025); annotated metabolites were then mapped to KEGG Pathway database (http://www.kegg.jp/kegg/pathway.html, accessed on 15 March 2025).

### 2.8. Statistical Analysis

The raw data were processed based on information from the Kyoto Encyclopedia of Genes and Genomes database (KEGG, https://www.genome.jp/kegg/pathway.html, accessed on 9 January 2025) and the Human Metabolome Database (HMDB, https://www.hmdb.ca/, accessed on 9 January 2025) for compound annotation. Unsupervised and supervised dimensionality reduction methods were utilized to conduct principal component analysis (PCA) and orthogonal partial least squares discriminant analysis (OPLS-DA) using the R.4.4.2 (http://www.r-project.org/, accessed on 15 July 2025). Use the R pheatmap software (v1.0.12) [35] to create a hierarchical cluster (HCA) heatmap after the z-score normalization of metabolites in all samples. Differential metabolites were identified based on the variable weight value (VIP) and *p* value from the (O)PLS model, with metabolites having VIP > 1 and *p* < 0.05 considered as differential. Correlation and significance analysis of data on differential metabolites (DMs) and antioxidant activity were performed using SPSS 27.0 software.

### 2.9. Lighting System Configuration and Calibration

A programmable LED incubator (Model: ZQZY-AGS8, Zhichu, Shanghai, China) was employed for the cultivation experiments. The lighting system was configured and calibrated according to the following procedures to ensure accuracy and consistency of photometric parameters.

#### 2.9.1. Light Source and Spectrum

The incubator was equipped with an overhead LED panel emitting a continuous full spectrum that simulates solar radiation. The spectrum exhibited characteristic peaks at 450 nm (blue light) and 660 nm (red light).

#### 2.9.2. Validation of Light Intensity and Uniformity

Light intensity was quantified as photosynthetic photon flux density (PPFD, unit: μmol·m^−2^·s^−1^). All PPFD measurements were conducted using a PQS-1 photosynthetic active radiation sensor (Beijing Ecotech Technology Co., Ltd., Beijing, China). Prior to introducing experimental samples, a 5 × 5 grid (25 measurement points) was established across the target cultivation plane (shelf positioned 15 cm below the light source), covering the entire effective cultivation area. After operating the lighting system for 30 min to achieve stable output, PPFD values were recorded at each point sequentially. The calculated mean PPFD was 150 ± 5 μmol·m^−2^·s^−1^, consistent with the experimental target. The spatial uniformity of light intensity, expressed as U_0_ (defined as the ratio of minimum to mean PPFD), was determined to be 0.78, indicating acceptable homogeneity across the cultivation area.

#### 2.9.3. Photoperiod Control

A built-in programmable timer regulated the photoperiod, which was set to a 12 h light/12 h dark cycle. All experiments were performed under the validated lighting conditions described above.

## 3. Results

### 3.1. Comparative Analysis of Active Substances and Antioxidant Activities Among Different Photoperiod Groups

**Figure 1:**
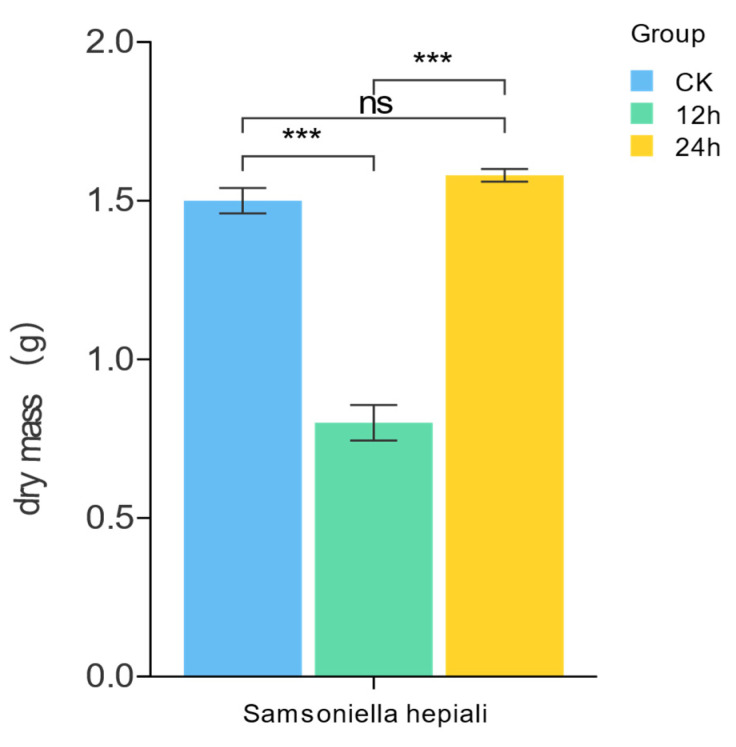
Light-duration treatments on the dry weight of *Samsoniella hepiali* Mycelium (***: *p* < 0.001, ns: no significant difference).

**Figure 2:**
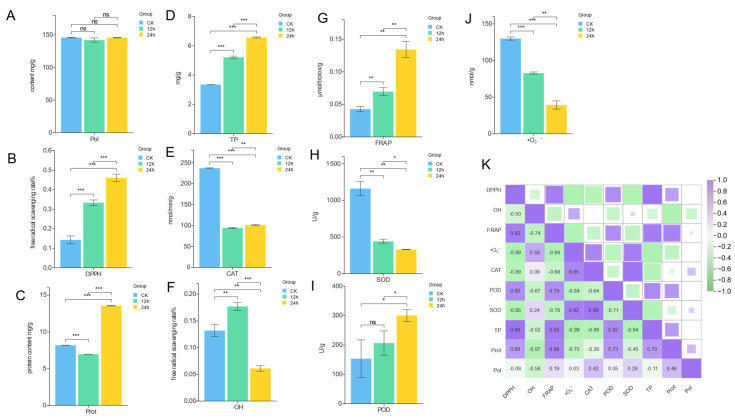
Comparative analysis of active substance contents and antioxidant activities among different groups (**A**–**J**), (***: *p* < 0.001, **: *p* < 0.01, *: *p* < 0.05, ns: no significant difference) and correlation analysis between active substance contents and antioxidant activities (**K**), Purple indicates a positive correlation, and green represents/indicates a negative correlation.

*S. hepiali* exhibits significant pharmacological effects similar to those of Cordyceps sinensis, mainly attributed to its antioxidant properties. In this study, the antioxidant capacity of *S. hepiali* under different light durations was comprehensively evaluated by measuring the activities of DPPH, CAT, POD, and SOD enzymes, the contents of ·OH and ·O_2_^−^, the total antioxidant capacity of FRAP, as well as the contents of active substances such as polysaccharides (Pol), proteins (Prot), and total phenols (TP) (Figure 1 and Figure 2).

There was no significant difference in dry weight between the CK group and the 24 h group, while the dry weight of the 12 h group was significantly lower than that of the CK group and the 24 h group (*p* < 0.001). There was no significant difference in polysaccharide content among all groups. However, differences were observed in FRAP value, POD, and DPPH among the groups as follows: the 24 h group showed stronger antioxidant capacity in these indicators, followed by the 12 h group, and the CK group was the lowest.

The SOD result of the CK group was extremely significantly higher than those of the previous two groups (*p*< 0.001). In the 24 h group, the SOD activity was significantly lower than CK (*p* < 0.001) and lower than 12 h (*p* < 0.01).

A lower content of ·O_2_^−^ indicates a stronger ability of the sample to scavenge superoxide anion ·O_2_^−^. The content of ·O_2_^−^ was the highest in the CK group, slightly lower in the 12 h group, and the lowest in the 24 h group. These results indicate that *S. hepiali* under 24 h light treatment had the strongest ability to scavenge superoxide anion ·O_2_^−^, followed by the 12 h light treatment, while the scavenging ability in the CK group without light treatment was relatively the weakest. This result is consistent with the antioxidant capacity trends reflected by previous indicators such as FRAP value, POD, and DPPH, further indicating that a longer light duration is beneficial to enhancing the antioxidant capacity of *S. hepiali*.

To investigate the relationship between functional metabolites and antioxidant properties, a correlation heatmap was generated (Figure 2K). The analysis revealed that total phenolic content (TP) exhibited a highly significant positive correlation with both DPPH radical scavenging capacity and FRAP ferric reducing ability, as well as a significant positive correlation with peroxidase (POD) activity. In contrast, TP showed a highly significant negative correlation with superoxide anion radical (·O_2_^−^) levels, suggesting that TP accumulation may be a key factor in enhancing the antioxidant capacity of *S. hepiali*. Protein content (Prot) was found to be highly significantly negatively correlated with hydroxyl radical (·OH) but significantly positively correlated with FRAP values, indicating that Prot accumulation also contributes to the antioxidant activity of the samples. Notably, polysaccharide content (Pol) showed only weak correlations with the antioxidant indices mentioned above. Furthermore, TP was negatively correlated with both superoxide dismutase (SOD) and catalase (CAT) activities, further highlighting the complex interactions between metabolic components and bioactivity in *S. hepiali*.

### 3.2. Identification of Differentially Accumulated Metabolites Among Comparative Groups Under Varying Photoperiods

Owing to significant phenotypic divergence observed among strains cultivated under distinct photoperiodic regimes, we conducted comparative metabolomic profiling of control (CK), 12 h light (12 h), and continuous light (24 h) groups to delineate light-driven metabolic reprogramming. Quality control (QC) samples from the three cohorts were analyzed on an UPLC–Q-Exactive Plus MS platform to assess analytical reproducibility and stability. The overlay of extracted ion chromatograms revealed highly consistent peak intensities and retention times across QC injections, indicating minimal instrumental drift and thus confirming the reliability of the acquired dataset.

**Figure 3:**
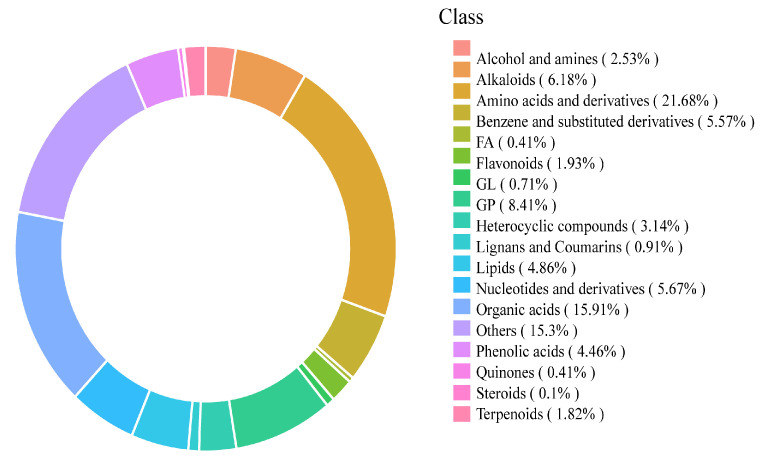
Comparison of total ion chromatograms (TICs) across sample groups.

**Figure 4:**
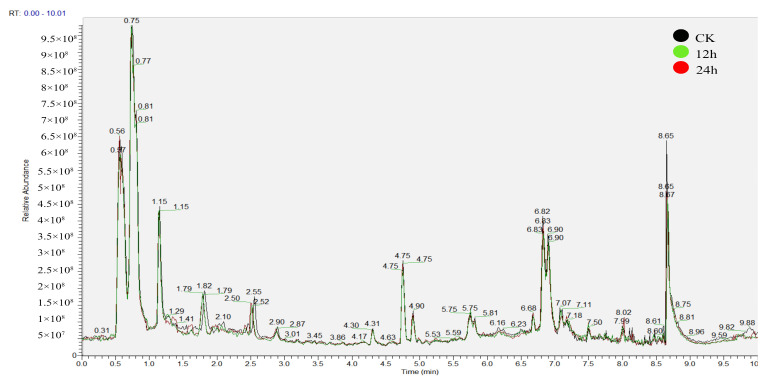
Metabolite classification circular diagram.

A total of 3643 non-redundant metabolites were confidently annotated from the tri-group cohorts, comprising 2804 features detected in positive electrospray ionization mode (ESI^+^) and 839 in negative mode (ESI^−^) (Figure 3). Metabolite classification according to the ClassyFire ontology assigned these entities to 18 super-classes: alcohols and polyols, amines, alkaloids, amino acids and derivatives, benzenoids, fatty acyls, flavonoids, glycerolipids, glycerophospholipids, heterocyclic compounds, lignans and coumarins, prenol lipids, nucleosides and nucleotides, organic acids, quinones, phenolic acids, steroids, terpenoids, and unclassified metabolites. Amino acid derivatives constituted the most abundant super-class (21.68%), followed by organic acids (15.91%), underscoring their central roles in light-dependent metabolic remodeling (Figure 4).

**Figure 5:**
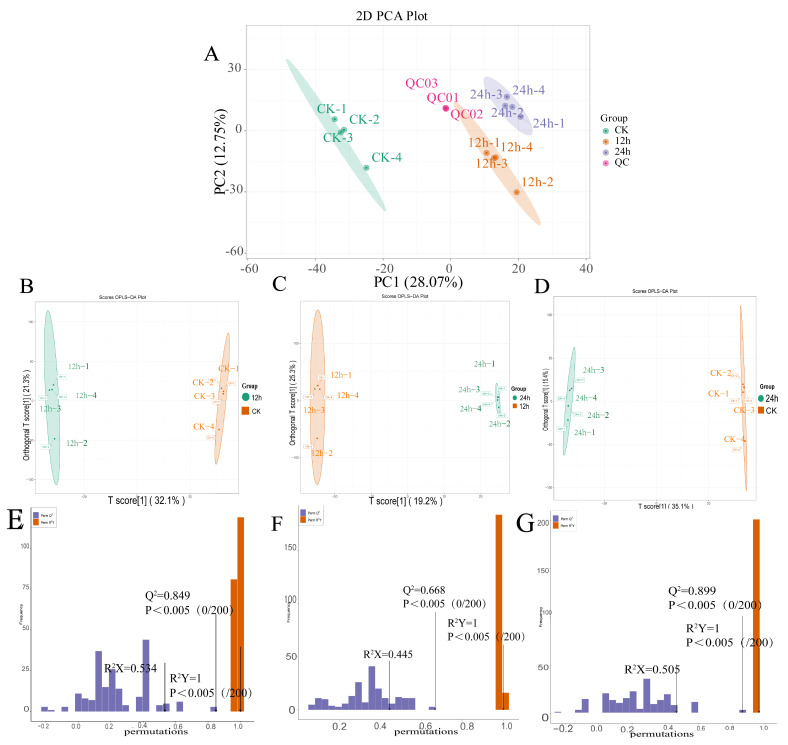
Scatter plot of PC1 vs. PC2 from PCA (**A**), and OPLS-DA score plots together with permutation tests for the pairwise comparisons CK vs. 12 h (**B**,**E**), CK vs. 24 h (**C**,**F**), and 12 h vs. 24 h (**D**,**G**).

Principal component analysis (PCA) was conducted to visualize the metabolomic profiles of the CK, 12 h, 24 h, and quality-control (QC) samples. The PCA scores plot revealed a clear segregation among the three biological groups, accompanied by moderate intra-group variability. PC 1 explained 12.75% of the total variance, whereas PC 2 accounted for 28.07%. Each sample cluster exhibited strong cohesion and high reproducibility, and an unambiguous separation trend was observed between groups, indicating significant compositional differences among CK, 12 h, and 24 h samples. QC samples clustered tightly at the centroid of the plot, demonstrating minimal analytical drift and confirming the robustness of the analytical platform and the reliability of the acquired metabolomic data (Figure 5).

Orthogonal partial least-squares discriminant analysis (OPLS-DA) further validated these observations. In every pairwise model, the two compared sample groups were clearly resolved within their respective 95% confidence ellipses. The model quality parameters satisfied stringent criteria for reliability (R^2^Y = 1, Q^2^ > 0.5).

In the OPLS-DA model, the VIP of the first principal component was used as an indicator of the influence intensity of each metabolite content on samples. A method combining *t*-test with a significance level of *p* < 0.005 and VIP > 1 was adopted to identify differential metabolites and auxiliary markers between different groups, assisting the metabolite screening process. Hierarchical cluster analysis (HCA) based on the top 25 metabolites showed that different metabolites were grouped together, and the clustering results indicated differences in metabolite expression between samples.

**Figure 6:**
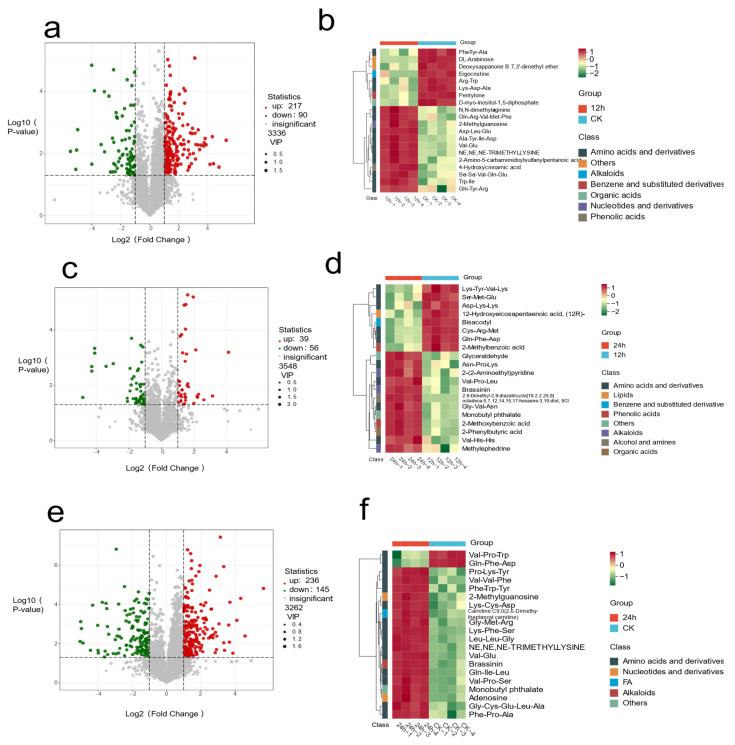
Volcano plots for pairwise comparisons CK vs. 12 h (**a**), CK vs. 24 h (**c**) and 12 h vs. 24 h (**e**). Metabolites significantly up-regulated are colored red, and those significantly down-regulated are colored green. Hierarchical clustering heat-maps for the same pairwise comparisons CK vs. 12 h (**b**), CK vs. 24 h (**d**) and 12 h vs. 24 h (**f**).

Differential metabolites were visually represented in the volcano plot (Figure 6). It was found that a total of 217 metabolites were up-regulated and 90 were down-regulated between the 12 h and CK samples; a total of 236 metabolites were up-regulated and 145 were down-regulated between the 24 h and CK samples; and 39 were up-regulated and 56 were down-regulated between the 12 h and 24 h samples.

The Venn diagram (Table 1) showed that seventeen overlapping DMs among CK, 12 h, and 24 h groups could be further divided into specific known DMs, including monocrotaline, 3-indolepropionic acid, brassinin, N’-(2-aminophenyl)-N-(4-methylphenyl)heptanediamine, prolyl-arginyl-asparagine, phenylalanyl-prolyl-threonine, L-leucyl-L-isoleucyl-L-tyrosine, phenylalanyl-valyl-cysteine, histidyl-phenylalanyl-phenylalanine, 2-(2-methylphenoxy)-N-[2-(4-methylphenyl)-2H-benzotriazol-5-yl]acetamide, (3-beta,4-alpha)-3,23-dihydroxyolean-12-en-28-oic acid, Ile-Ile-Arg-Ser-Ser, PE-NMe2(14:1(9Z)/22:1(13Z)), (2R,4S)-2,4-diaminopentanoate; D-threo-2,4-diaminopentanoate; 2,4-diaminopentanoate, threonyl-arginyl-lysine, 2-phenylbutyric acid, and L-tyrosyl-L-proline. Therefore, these DMs can serve as potential biomarkers for distinguishing ck, 12 h, and 24 h groups.

Among these differential metabolites, we further conducted visualization analysis, and the results showed that these metabolites could be divided into six subgroups. The results indicated that amino acids and their derivatives (prolyl-arginyl-asparagine, phenylalanyl-prolyl-threonine, L-leucyl-L-isoleucyl-L-tyrosine, phenylalanyl-valyl-cysteine, histidyl-phenylalanyl-phenylalanine), organic acids [(3-beta,4-alpha)-3,23-dihydroxyolean-12-en-28-oic acid, 2-(2-methylphenoxy)-N-[2-(4-methylphenyl)-2H-benzotriazol-5-yl]acetamide], and glycerophospholipid metabolite [PE-NMe2(14:1(9Z)/22:1(13Z))] had the highest expression abundance in the CK group. Benzene and its derivatives (N’-(2-aminophenyl)-N-(4-methylphenyl) heptanediamine) had a higher content in the 24 h group, while indole alkaloids (3-indolepropionic acid, brassinin, monocrotaline) in the 12 h group showed higher expression abundance. This provides valuable insights for identifying potential biomarkers.

### 3.3. KEGG Pathway Analysis of Differentially Accumulated Metabolites Among Comparative Groups Under Varying Photoperiods

**Figure 7:**
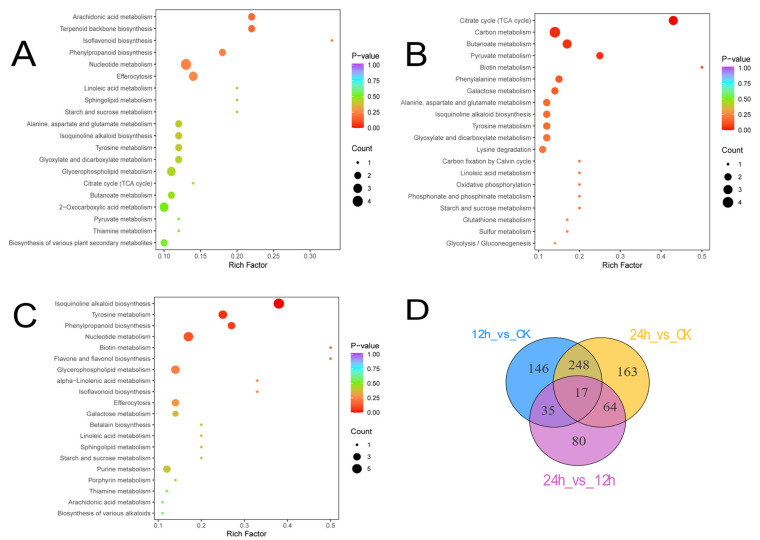
Bubble plots of KEGG pathway enrichment (top 20) for the pairwise comparisons CK vs. 12 h (**A**), CK vs. 24 h (**B**) and 12 h vs. 24 h (**C**), Count: Number of significantly differential metabolites found in the pathway. Venn diagram (**D**).

Using the KEGG metabolic pathway database (Figure 7) and the numbers of differential metabolites (DMs) in CK, 12 h, and 24 h, the top 20 pathways with the highest enrichment ratios were selected for joint analysis. Isoquinoline alkaloid biosynthesis, the citrate (TCA) cycle, and arachidonic acid metabolism ranked as the three most enriched pathways; linoleic acid metabolism and tyrosine metabolism were commonly enriched across all comparison pairs. Eleven pathways were jointly enriched between CK vs. 12 h and CK vs. 24 h. In the 12 h vs. 24 h comparison, citrate cycle, carbon metabolism, and phenylpropanoid biosynthesis were significantly enriched.

**Figure 8:**
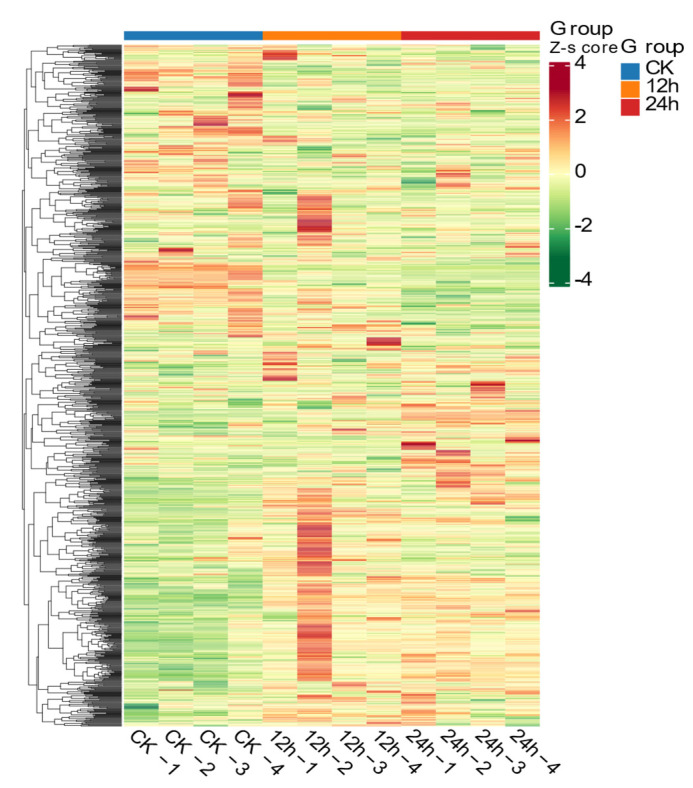
Hierarchical clustering of DMs identified among the CK_12h_24h comparison groups. Red indicates higher relative expression; green indicates lower relative expression.

To evaluate the rationality of the candidate metabolites and to provide a comprehensive and intuitive visualization of the inter-sample relationships as well as the differential expression patterns of metabolites across different samples, we performed hierarchical clustering analysis (Figure 8) based on the abundance of qualitatively significant differential metabolites. The results demonstrated that the CK group exhibited a distinct metabolic profile, characterized by a substantial number of uniquely highly expressed (red) and lowly expressed (green) metabolites. The metabolic pattern of the 12 h group was intermediate between the CK and 24 h groups but clearly deviated from CK, indicating that 12 h light exposure was sufficient to initiate significant metabolic reprogramming. In contrast, the 24 h group showed the strongest contrast to the CK group, displaying a large number of metabolites with expression trends opposite those in CK.

### 3.4. Integrated Analysis

**Figure 9:**
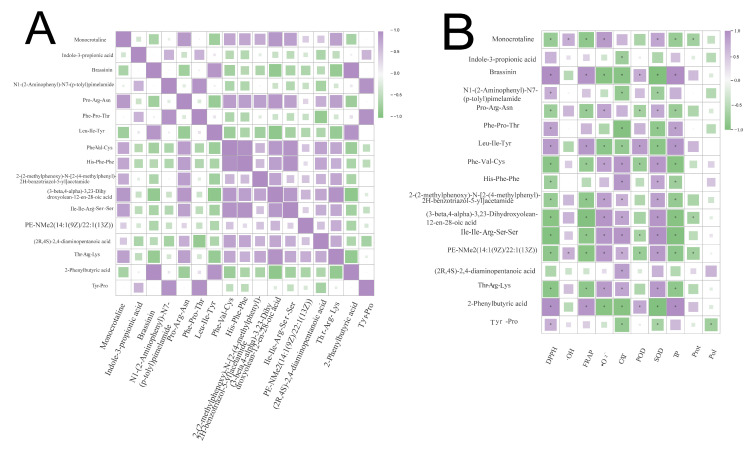
Correlation analysis among significantly enriched metabolites (**A**) and correlation analysis of metabolites with active substances and antioxidant activity (**B**), *: significant.

Mantel test and Pearson correlation analysis were employed to investigate the relationships between DPPH, FRAP, ·OH, CAT, ·O_2_^−^, POD, SOD, total phenols (TP), polysaccharides (Pol), proteins (Prot), and key differential metabolites (DMs). The results (Figure 9) showed that antioxidant activity was significantly correlated with various amino acids and their derivative DMs. The correlation heatmap indicated the correlations between most amino acid DMs and organic acid DMs. For example, 2-phenylbutyric acid and prolyl-arginyl-asparagine were positively correlated with DPPH· and FRAP and negatively correlated with ·O_2_^−^. They also showed a positive correlation with TP content, but a weak correlation with Pol and Protein contents, suggesting that changes in TP content are related to changes in the contents of these DMs.

Alcohols and amines: 2-(2-methylphenoxy)-N-[2-(4-methylphenyl)-2H-benzotriazol-5-yl] acetamide was positively correlated with FRAP. The abundance of this DM was consistent with the trends of DPPH· and FRAP observed in various samples.

DMs that showed a significant positive correlation with CAT and SOD included L-tyrosyl-L-proline, 2-phenylbutyric acid, brassinin, and L-leucyl-L-isoleucyl-L-tyrosine. The expression abundance of these DMs was negatively correlated with DPPH·, FRAP, and ·OH in different samples. These DMs mainly belong to amino acids, indicating that amino acids play a major role in determining the antioxidant activity of *S. hepiali*.

The ·O_2_^−^ content measured in this study was significantly negatively correlated with most DMs, which suggests that the scavenging rate of ·O_2_^−^ is positively correlated with other antioxidant indicators. However, ·OH and POD had weak correlations with other antioxidant indicators such as DPPH· and FRAP, and also showed weak correlations with other DMs.

## 4. Discussion

The medicinal value of *S. hepiali* primarily stems from its antioxidant capacity, which varies under different photoperiods. Antioxidant parameters and bioactive compound levels at 0 h, 12 h, and 24 h provide phenotypic evidence for subsequent non-targeted metabolomics analysis. The 24 h group showed the highest total phenolics, DPPH scavenging activity, FRAP value, and POD activity, along with the lowest superoxide anion level, indicating superior antioxidant performance. This may result from phenolic accumulation countering oxidative stress and synergistic upregulation of the enzymatic antioxidant system [33,34]. Correlation analysis supports the positive relationships among these indicators. Protein content was highest in the 24 h group, followed by 0 h and 12 h, suggesting photoperiod-dependent metabolic adaptation. SOD and CAT activities were highest in the dark control group, while hydroxyl radical scavenging peaked in the 12 h group, reflecting distinct light-responsive metabolic traits. We hypothesize that exposure to light stress elicits a dual response in *S. hepiali*: while it upregulates antioxidant capacity and enhances the production of most secondary metabolites, the concomitant decline in specific compounds points to potential resource competition or regulatory antagonism among biosynthetic pathways under photostress. Functioning as a systemic regulator, light activates broad-spectrum antioxidant defenses while selectively suppressing particular metabolic routes responsible for the synthesis of the diminished compounds. This regulatory divergence suggests a heterogeneous distribution of light-responsive genetic elements within the *S. hepiali* metabolic network, leading to pathway-specific expression patterns in response to uniform environmental stimuli. The observed reduction in selected metabolites may thus represent a strategic trade-off, offering crucial insights into the fungus’ distinctive adaptative response to light [35]. Notably, polysaccharide content showed no significant difference across groups, indicating that antioxidant variations are likely due to other components, possibly common saccharides such as glucosides, ribose, or mannose, which remain stable under varying light conditions [36]. The biomass of *S. hepiali mycelia* in the dark control group and the 24 h light group was relatively similar, whereas the biomass in the 12 h light group was significantly lower than both. It is preliminarily speculated that this may be related to the fungus’ requirement for a relatively stable growth environment, as continuously varying photoperiods may interfere with the accumulation of mycelial biomass. According to previous research, intermittent LED light exposure (i.e., 12 h of light followed by 12 h of darkness per day) promotes mycelial growth in *Pleurotus geesteranus* and enhances its ability to degrade selenium-fortified wheat bran and facilitate selenium biotransformation [29,37]. This finding appears to contradict the results of our study, and such discrepancies may be attributed to interspecific differences in biological responses to light [38]. Research by Siwulski et al. (2012) demonstrated that under light intensities of 500–700 lux, a daily photoperiod of 14 h maximized the yield and improved the fruiting body morphology of four *Pleurotus* species [31]. Wang et al. (2011) reported that both blue light and darkness accelerated the mycelial growth of *Ganoderma lucidum*, though the mycelial morphology under dark conditions was inferior to that under blue light [39]. A research also observed that blue light treatment ensured stable mycelial growth of *G. lucidum* throughout the cultivation period. In contrast, blue light suppressed mycelial growth of *Cordyceps militaris* in liquid culture [40]. Although these studies focused on specific light qualities (e.g., blue light) rather than full-spectrum light, they collectively indicate that the effects of light vary significantly across fungal species. Different light qualities exert varying regulatory effects on the activities of superoxide dismutase (SOD), catalase (CAT), and peroxidase (POD), as well as on the endogenous indole-3-acetic acid (IAA) levels in *Ganoderma lucidum* mycelia [40]. Studies have shown that blue light treatment effectively enhances the diversity and content of triterpenoid acids in *G. lucidum* mycelia and significantly promotes polysaccharide accumulation during the budding, cap-opening, and spore-releasing stages [41]. Furthermore, blue light also facilitates the growth of *Cordyceps militaris* and the synthesis of cordycepin in its stroma [42]. Research shows that the SOD activity in mature fruiting bodies under natural light was significantly higher than that under blue light, while no notable difference was observed in CAT activity. POD activity, however, varied depending on the fungal strain. Notably, blue light irradiation significantly increased carotenoid content, whereas adenosine and mannitol levels remained largely unaffected [43]. The contents of cordycepin and crude polysaccharides exhibited strain-specific responses. Further research indicated that during the submerged fermentation of *Pleurotus eryngii*, shorter wavelengths of light promoted polysaccharide accumulation, with the highest yield achieved under blue light [28]. Similarly, it was reported that blue LED irradiation increased the ergosterol and total polyphenol contents in *Hypsizygus marmoreus* fruiting bodies, accompanied by enhanced free-radical scavenging capacity [23]. Although variations in experimental design and species selection led to some inconsistencies across studies, the overall findings suggest that light exposure positively modulates the antioxidant capacity in various fungi, which aligns with the conclusions drawn in this study. Future research should expand the range of detected physiological indicators—such as adenosine and mannitol—and incorporate diverse lighting conditions to systematically elucidate the mechanisms underlying the effects of light on the physiological characteristics and antioxidant activity of *S. hepiali*. This study has certain limitations. Currently, there is limited research on the effects of light on filamentous fungi, with most studies focusing on the illumination of fruiting bodies. Furthermore, experimental conditions, such as light quality and intensity, vary considerably, which hinders direct comparability. Future research should build upon these findings to further investigate the influence of different light qualities and intensities on *S. hepiali*, or by combining these factors into a multifactorial experimental design.

In this study, a multi-database strategy was combined with LC-MS/MS and multivariate statistics to perform qualitative metabolomic profiling of three experimental groups. This workflow maintained ultrahigh sensitivity while shortening analytical run-time, yielding highly accurate qualitative data. Subsequent targeted quantification of antioxidant-relevant compounds clarified the differential metabolites (DMs) that govern the antioxidant capacity and bioactive inventory of *S. hepiali*, thereby establishing a foundation for deeper investigations into amino-acid metabolism. Metabolic-pathway mapping indicated that citrate-cycle biosynthesis, linoleate metabolism, and isoquinoline-alkaloid biosynthesis were the top three pathways responsible for the observed differences in antioxidant activity and bioactive-compound content. Further annotation revealed that the DMs were predominantly amino acids and derivatives, benzenoids, alcohols and amines, organic acids, glycerophospholipids, and nucleotides, consistent with previous reports [44]. Amino acids and their derivatives, the principal constituents across all three comparisons, are not only proteinogenic building blocks but also multifunctional mediators of diverse physiological processes. Numerous secondary metabolites within this class have validated therapeutic value. Glutathione, for example, sustains immune homeostasis while functioning as an antioxidant and detoxifying agent [45,46,47]. Lysine promotes growth, enhances immunity, exerts antiviral effects, facilitates lipid oxidation, and alleviates anxiety [48,49]; it also improves the absorption and synergistic action of other nutrients. Among organic acids, succinic acid is widely used in the food industry as a flavor enhancer, acidulant, and buffering agent in ham, sausages, seafood, and seasoning liquids; it also serves as a preservative, pH regulator, and solubilizer. Pharmacologically, it is a precursor for antidotes, diuretics, sedatives, hemostatics, and certain vitamins (A and B). In electroplating, succinate acts as a chelator to prevent metal etching and pitting, while in consumer goods it is formulated into detergents, soaps, demulsifiers, depilatories, toothpaste, and anti-aging cosmetics. In materials science it is employed as a lubricant additive and in textiles as an anti-shrink and dye-affinity agent [50,51,52,53].

KEGG enrichment analyses delineated the metabolic pathways differentially activated in *S. hepiali*. Between the dark control and illuminated groups (CK vs. 12 h, CK vs. 24 h), significantly enriched pathways included arachidonic-acid metabolism, phenylpropanoid biosynthesis, nucleotide metabolism, efferocytosis, alanine/aspartate/glutamate metabolism, and isoquinoline-alkaloid biosynthesis. In contrast, the comparison between the two illuminated groups (12 h vs. 24 h) was characterized by enrichment of the citrate cycle (TCA cycle), carbon metabolism, and butanoate metabolism. The comparative analysis between the control group (CK) and the 12 h light/dark cycle group (12 h) revealed significant enrichment of the TCA cycle and carbon metabolism pathways. This was accompanied by a reduction in mycelial biomass in the 12 h group, alongside an observed increase in hydroxyl radical scavenging capacity. These phenomena may be attributed to the persistent metabolic disruption induced by the alternating light/dark environment in *S. hepiali*. Under frequent light–dark transitions, the fungus must continuously shut down one set of metabolic pathways and activate another. This process of “metabolic reprogramming” is inherently energy-consuming and can lead to metabolic oscillations. In particular, during the transition from dark to light, the fungal light-sensing and energy conversion systems—still in a “dark-adapted” state—may fail to promptly process the sudden influx of light energy. This can cause over-reduction in the electron transport chain, resulting in substantial generation of reactive oxygen species (ROS), such as superoxide anions and hydrogen peroxide. Similarly, a rapid shift from light to dark may lead to the accumulation of reducing equivalents and metabolic intermediates, potentially also triggering ROS production. Thus, the 12 h periodic light–dark transitions subject the cells to repeated episodes of intense oxidative stress. To mitigate the resulting oxidative damage, the fungus must allocate substantial resources toward synthesizing antioxidants (e.g., glutathione, vitamins C and E) and repair damaged proteins, lipids, and DNA. This recurring “damage–repair” cycle diverts carbon and energy resources that would otherwise be directed toward mycelial growth and biomass accumulation, ultimately leading to a net reduction in biomass. This interpretation is further supported by the observation that most antioxidant indicators were higher in the 12 h group compared to the CK group. Notably, the highest hydroxyl radical scavenging rate was detected in the 12 h group, which may reflect a unique metabolic adaptation of *S. hepiali* under this specific photoperiod regime. Enhancing the capacity to scavenge hydroxyl radicals—the most destructive ROS—could represent a crucial physiological strategy to alleviate oxidative stress induced by periodic light–dark transitions [54,55].

Arachidonic acid, a membrane phospholipid constituent, serves as a precursor for prostaglandins and leukotrienes. Via cyclo-oxygenase and lipoxygenase cascades, it generates spasmogenic mediators during immune responses. Adequate dietary arachidonate reduces essential-amino-acid catabolism and confers radioprotective effects on skin; its intracellular accumulation may therefore reflect metabolic vigor [56,57,58]. Phenylpropanoid biosynthesis yields the largest flavonoid subclass, whose C6-C3-C6 scaffold (two phenolic rings linked by a three-carbon bridge) gives rise to flavones, flavonols, flavanones, isoflavones, anthocyanins, proanthocyanidins, and tannins. These non-essential dietary constituents add nutritional value through anti-inflammatory, antimicrobial, and anticancer activities; their primary benefit is antioxidant scavenging of reactive oxygen species (ROS), although the underlying molecular mechanisms remain incompletely understood [59,60,61,62]. Notably, arachidonic-acid metabolism and phenylpropanoid biosynthesis were enriched only in CK vs. 12 h and CK vs. 24 h comparisons, whereas the TCA cycle and carbon metabolism were jointly enriched in 12 h vs. 24 h. Meanwhile, a reductive TCA cycle operates in certain microbes for carbon fixation. Furthermore, butyrate derived from butanoate metabolism acts as a signaling molecule by binding G-protein-coupled receptors (GPCRs) or by inhibiting histone deacetylases (HDACs), thereby regulating lipid metabolism and implying a lipid-lowering capacity of *S. hepiali* under light. Thus, the accumulation of TCA-cycle, carbon-metabolism, and butanoate-metabolism intermediates under illuminated conditions may constitute the key metabolic determinants of the observed inter-group differences.

Correlation analysis further revealed a weak association between polysaccharide/protein contents and most DMs, implying that polysaccharide and protein metabolism constitute relatively independent branches within the global metabolic network. Consequently, their accumulation appears to be regulated separately from the reprogramming of low-molecular-weight metabolites when *S. hepiali* is exposed to different photoperiods. From a systems perspective, the identified DMs form an intricate regulatory network. Amino acids and derivatives, while numerically dominant, are also pivotal for antioxidant activity, presumably by modulating intracellular redox status through participation in multiple biochemical reactions. Organic acids, benzenoids, and other chemical classes further integrate into this network, collectively shaping the physiological and metabolic phenotypes under varying light regimes.

## 5. Conclusions

This study investigated the effects of different photoperiods on the antioxidant capacity and bioactive components of *Samsoniella hepiali*. Comparative analysis revealed that continuous illumination (24 h) resulted in the highest protein content, total phenolic (TP) content, DPPH radical scavenging activity, FRAP value, and peroxidase (POD) activity, along with the lowest superoxide anion (·O_2_^−^) concentration, indicating a comprehensively enhanced antioxidant profile. In contrast, the 12 h group exhibited the highest hydroxyl radical (·OH) level but the lowest dry mycelial biomass. The analysis revealed that total phenolic content (TP) exhibited a highly significant positive correlation with both DPPH radical scavenging capacity and FRAP ferric reducing ability, as well as a significant positive correlation with peroxidase (POD) activity. In contrast, TP showed a highly significant negative correlation with superoxide anion radical (·O_2_^−^) levels, suggesting that TP accumulation may be a key factor in enhancing the antioxidant capacity of *S. hepiali*. Protein content (Prot) was found to be highly significantly negatively correlated with hydroxyl radical (·OH) but significantly positively correlated with FRAP values, indicating that Prot accumulation also contributes to the antioxidant activity of the samples. Notably, polysaccharide content (Pol) showed only weak correlations with the antioxidant indices mentioned above. Furthermore, TP was negatively correlated with both superoxide dismutase (SOD) and catalase (CAT) activities, further highlighting the complex interactions between metabolic components and bioactivity in *S. hepiali*.

Non-targeted metabolomics analysis of mycelia under dark (CK), 12 h light, and 24 h light conditions identified a total of 3643 metabolites. Comparative analysis showed 217 up-regulated and 90 down-regulated metabolites in the 12 h light group compared to CK, 236 up-regulated and 145 down-regulated in the 24 h light group compared to CK, and 39 up-regulated and 56 down-regulated between the 12 h and 24 h light groups. These differential metabolites may serve as potential biomarkers for distinguishing between conventional and light-treated strains. Correlation analysis indicated that amino acid/organic acid derivatives were positively correlated with DPPH radical scavenging capacity, FRAP value, and total phenolic content, but negatively correlated with superoxide anion (·O_2_^−^) concentration. Alcohol and amine derivatives also showed significant positive correlations with antioxidant indices. KEGG pathway analysis revealed that eleven metabolic pathways were commonly enriched in both the CK vs. 12 h and CK vs. 24 h comparisons, including phenylpropanoid biosynthesis, isoflavonoid biosynthesis, nucleotide metabolism, efferocytosis, linoleic acid metabolism, sphingolipid metabolism, starch and sucrose metabolism, glycerophospholipid metabolism, the citrate cycle (TCA cycle), thiamine metabolism, and arachidonic acid metabolism. In the 12 h vs. 24 h light comparison, the citrate cycle (TCA cycle), carbon metabolism, and phenylpropanoid biosynthesis pathways were significantly enriched.

## Figures and Tables

**Table 1:** 17 overlapping differential metabolites.

Compounds	Class I	Class II	Formula	Level	Score
Monocrotaline	Alkaloids	Pyrrole alkaloids	C_16_H_23_NO_6_	2	0.7677
Indole-3-propionic acid	Alkaloids	Plumerane	C_11_H_11_NO_2_	3	0.7800
Brassinin	Alkaloids	Plumerane	C_11_H_12_N_2_S_2_	3	0.6460
N1-(2-Aminophenyl)-N7-(p-tolyl)pimelamide	Benzene and substituted derivatives	Benzene and substituted derivatives	C_20_H_25_N_3_O_2_	3	0.5215
Pro-Arg-Asn	Amino acids and derivatives	Amino acids and derivatives	C_15_H_27_N_7_O_5_	2	0.7665
Phe-Pro-Thr	Amino acids and derivatives	Amino acids and derivatives	C_18_H_25_N_3_O_5_	2	0.7623
Leu-Ile-Tyr	Amino acids and derivatives	Amino acids and derivatives	C_21_H_33_N_3_O_5_	2	0.7605
Phe-Val-Cys	Amino acids and derivatives	Amino acids and derivatives	C_17_H_25_N_3_O_4_S	2	0.7175
His-Phe-Phe	Amino acids and derivatives	Amino acids and derivatives	C_24_H_27_N_5_O_4_	2	0.7110
2-(2-methylphenoxy)-N-[2-(4-methylphenyl)- 3-2H-benzotriazol-5-yl]acetamide	Alcohol and amines	Amines	C_22_H_20_N_4_O_2_	3	0.7272
(3-beta,4-alpha)-3,23-Dihydroxyolean-12-en-28-oic acid	Organic acids	Organic acids	C_30_H_47_O_4_^−^	3	0.6630
Ile-Ile-Arg-Ser-Ser	Amino acids and derivatives	Amino acids and derivatives	C_24_H_46_N_8_O_8_	3	0.6073
PE-NMe2(14:1(9Z)/22:1(13Z))	GP	PE	C_43_H_82_NO_8_P	3	0.6022
(2R,4S)-2,4-diaminopentanoic acid	Organic acids	Organic acids	C_5_H_12_N_2_O_2_	3	0.5962
Thr-Arg-Lys	Amino acids and derivatives	Amino acids and derivatives	C_16_H_33_N_7_O_5_	3	0.5096
2-Phenylbutyric acid	Organic acids	Organic acids	C_10_H_12_O_2_	3	0.8659
Tyr-Pro	Amino acids and derivatives	Amino acids and	C_14_H_18_N_2_O_4_	3	0.6357

## Data Availability

The supporting data for the findings of this study are available from the corresponding authors upon reasonable request.

## Abbreviations

DMs	Differential Metabolites
FRAP	total antioxidant capacity
·OH	hydroxyl free radical scavenging capacity
LC-MS/MS	high-performance liquid chromatography-tandem mass spectrometry
KEGG	Kyoto Encyclopedia of Genes and Genomes database
R^2^Y	Model (for Y-variable dataset) Interpretability
Q^2^	Model predictability
R^2^	Model Interpretability
ESI+	Positive Electrospray Ionization
ESI−	Negative Electrospray Ionization
OPLS-DA	Orthogonal partial least squares discriminant analysis
PCA	Principal Component Analysis
PC1	Principal Component 1
PC2	Principal Component 2
QC	quality control
ROS	Reactive oxygen species
·O_2_^−^	Superoxide anion radical scavenging capacity
TIC	total ion chromatograms
Pol	polysaccharide
Prot	protein
TP	total Phenolic Compounds
DPPH	1,1-Diphenyl-2-picrylhydrazyl
SOD	superoxide dismutase
POD	peroxidase
CAT	catalase
